# Does updating improve the methodological and reporting quality of systematic reviews?

**DOI:** 10.1186/1471-2288-6-27

**Published:** 2006-06-13

**Authors:** Beverley Shea, Maarten Boers, Jeremy M Grimshaw, Candyce Hamel, Lex M Bouter

**Affiliations:** 1 Community Information and Epidemiological Technologies (CIET), Ottawa, Ontario, Canada and VU University Medical Center, Amsterdam, The Netherlands; 2 Professor of Clinical Epidemiology and Director, Department of Clinical Epidemiology and Biostatistics, VU University Medical Center, Amsterdam, The Netherlands; 3 Professor and Director, Clinical Epidemiology Program, Ottawa Health Research Institute, Department of Medicine, University of Ottawa, Ottawa, Ontario, Canada; 4 Community Information and Epidemiological Technologies (CIET), Ottawa, Ontario, Canada; 5 Professor of Epidemiology and Director, Institute for Research in Extramural Medicine (EMGO Institute), VU University Medical Center, Amsterdam, The Netherlands

## Abstract

**Background:**

Systematic reviews (SRs) must be of high quality. The purpose of our research was to compare the methodological and reporting quality of original versus updated Cochrane SRs to determine whether updating had improved these two quality dimensions.

**Methods:**

We identifed updated Cochrane SRs published in issue 4, 2002 of the Cochrane Library. We assessed the updated and original versions of the SRs using two instruments: the 10 item enhanced Overview Quality Assessment Questionnaire (OQAQ), and an 18-item reporting quality checklist and flow chart based upon the Quality of Reporting of Meta-analyses (QUOROM) statement. At least two reviewers extracted data and assessed quality. We calculated the percentage (with a 95% confidence interval) of 'yes' answers to each question. We calculated mean differences in percentage, 95% confidence intervals and p-values for each of the individual items and the overall methodological quality score of the updated and pre-updated versions using OQAQ.

**Results:**

We assessed 53 SRs. There was no significant improvement in the global quality score of the OQAQ (mean difference 0.11 (-0.28; 0.70 p = 0.52)). Updated reviews showed a significant improvement of 18.9 (7.2; 30.6 p < .01) on the OQAQ item assessing whether the conclusions drawn by the author(s) were supported by the data and/or analysis presented in the SR. The QUOROM statement showed that the quality of reporting of Cochrane reviews improved in some areas with updating. Improvements were seen on the items relating to data sources reported in the abstract, with a significant difference of 17.0 (9.8; 28.7 p = 0.01), review methods, reported in the abstract 35 (24.1; 49.1 p = 0.00), searching methods 18.9 (9.7; 31.6 p = 0.01), and data abstraction 18.9 (11.7; 30.9 p = 0.00).

**Conclusion:**

The overall quality of Cochrane SRs is fair-to-good. Although reporting quality improved on certain individual items there was no overall improvement seen with updating and methodological quality remained unchanged. Further improvement of quality of reporting is possible. There is room for improvement of methodological quality as well. Authors updating reviews should address identified methodological or reporting weaknesses. We recommend to give full attention to both quality domains when updating SRs.

## Background

A number of papers have been published on the methodological and reporting quality of reviews. A review of 86 English language meta-analyses published between 1950 and 1986 by Sacks [1] assessed every report on fourteen items covering six content areas believed to be critical in the reporting of meta-analysis. Only 28% of these meta-analyses were found to address all six content areas. An updated survey in 1996 showed little change [2]. Shea [3] compared the methodological quality of paper-based and, electronic systematic reviews and found little difference and a lot of room for improvement. Assendelft [4] reviewed 51 reviews and noted that reviews that favoured a given intervention tended to have higher methodological quality scores. In contrast, Jadad and McQuay [5] reviewed 80 systematic reviews published between 1980 and 1992 and found a disconcerting link between reviews whose results favoured an intervention and poor methodological quality. Jadad found that Cochrane reviews had greater methodological rigor, more frequent updates and higher overall quality scores than those published in peer-reviewed paper journals, though both types were found to contain extensive and serious flaws.

The Cochrane Collaboration [6] is an international not-for-profit organization that conducts and updates systematic reviews of healthcare studies. With the exception of a few studies completed by Jadad [7,8] and Shea [9], little is known about the quality of Cochrane reviews and whether their quality is improving over time and with updating. To our knowledge, the impact of updating on their quality has not been evaluated nor studied.

Studies of the quality of systematic reviews can focus on methodological or reporting quality. Methodological quality is concerned with how well a systematic review was designed and conducted (e.g. literature searching, pooling of data, etc.). Reporting quality considers how well systematic reviewers have reported their methodology and findings.

The purpose of our study was to compare the methodological and reporting quality of Cochrane systematic reviews with that of their updated versions in order to determine whether updating contributed significantly to the improvement of their quality in these two dimensions.

## Methods

We selected all updated Cochrane systematic reviews from the Cochrane Database of Systematic Reviews 2002 [10], and the same reviews prior to their update. Updated reviews were chosen following the definition of 'updating' included in the Cochrane handbook [11].

Based on a previously published study [12], two instruments chosen to assess the quality of Cochrane systematic reviews for this study were the enhanced Overview Quality Assessment Questionnaire (OQAQ) scale [12-14] (Additional File 1) and the Quality of Reporting of Meta-analsyses (QUOROM) checklist [15] (Additional File 2).

The OQAQ was selected because it has strong face validity, provided data on several essential elements of its development, and had a published assessment of its construct validity available [13]. In addition, its validity has been thoroughly tested and established using a number of independent measures [14]. However, we noted difficulty applying the questions so we developed an enhanced version of the OQAQ which incorporated guidelines for its' use [12]. The OQAQ scale measures across a continuum using nine questions (items 1–9) designed to assess various aspects of the methodological quality of systematic reviews and one overall assessment question (item 10). When the scale is applied to a systematic review, the first nine items are scored by selecting either yes, no, partial/can't tell. The tenth item requires assessors to assign an overall quality score on a 7-point scale [13].

The QUOROM statement was chosen for assessing reporting quality. Although, this checklist has not yet been fully validated, extensive work has been conducted and reported [15]. The QUOROM statement is comprised of a checklist and flow diagram and was developed using a consensus process designed to strengthen the reliability of the estimates it yields when applied by different assessors. It estimates the overall reporting quality of systematic reviews. The checklist asks whether authors have provided readers with information on 18 items, including searches, selection, validity assessment, data abstraction, study characteristics, quantitative data syntheses and trial flow. It also asks whether authors have included a flow diagram with information about the number of randomized controlled trials identified, included and excluded, and the reasons for any exclusion. Individual checklist items included in this instrument are answered either yes, no and partial/can't tell [15].

For each included Cochrane systematic review, we calculated the percentage (with a 95% confidence interval) of 'yes' answers to each item of OQAQ and QUOROM. Individual Cochrane systematic reviews were then compared to their updated versions with respect to the percentage of 'yes' ratings received. A percentage difference (with a 95% confidence interval and p-values) of 'yes' answers was calculated for each individual question. In addition, a mean difference (with a 95% confidence interval and p-value) was calculated for the overall quality score (OQAQ) of the updated and pre-updated versions.

Assessments of all individual reviews were conducted independently by at least two assessors. One of these assessors (CH) had been involved with the Cochrane Collaboration for six years. The other assessor (BS) had been involved with the Cochrane Collaboration for ten years, and had carried out a number of systematic reviews. A third assessor (DF) was available to assist in the resolution of assessment discrepancies.

## Results

In total, 53 reviews were included. The mean period between the publication of the original and updated versions of the reviews was 2.7 years (range four months to five years). Complete results for both instruments applied to the reviews are provided in Tables 1 and 2, but highlights are summarized below.

**Table 1:** Comparisons of percent 'yes' and percent 'differences' using the enhanced Overview Quality Assessment Questionnaire (OQAQ).

**Questions OQAQ**	**Original Reviews yes, % reviews yes ** **[95% CI]**	**Updated Reviews yes, % reviews yes ** **[95% CI]**	**Difference % Yes ** **[95% CI] p-value**
1. Were the search methods used to find evidence reported?	81% [68.9 to 93.4]	87% [75.7 to 97.9]	5.7% [-6.6 to 17.9] 0.43
2. Was the search strategy for evidence reasonably comprehensive?	68% [47.0 to 88.7]	70% [48.3 to 91.3]	1.9% [-19.0 to 22.8] 0.83
3. Were the criteria used for deciding which studies to include in the overview reported?	98% [94.4 to 100.0]	91% [80.5 to 100.0]	-7.6% [-11.2 to 3.9] 0.09
4. Was bias in the selection of studies avoided?	64% [45.4 to 82.8]	70% [52.0 to 87.6]	5.7% [-13.1 to 24.4] 0.53
5. Were criteria used for assessing validity of the included studies reported?	89% [73.7 to 100.0]	96% [85.9 to 100.0]	7.6% [-7.4 to 22.5] 0.14
6. Was the validity of all studies referred to in the text assessed using appropriate criteria (either in selecting studies for inclusion or in analyzing studies that are cited)?	85% [70.6 to 99.2]	93% [82.9 to 100.0]	7.6% [-0.68 to 21.9] 0.22
7. Were methods used to combine the findings of relevant studies (to reach a conclusion) reported?	89% [66.2 to 99.8]	83% [71.5 to 100.0]	-5.7% [-022.9 to 11.6] 0.40
8. Were findings of the relevant studies combined appropriately relative to the primary question addressed?	87% [68.4 to 100.0]	91% [78.7 to 100.0]	3.8% [-14.6 to 22.2] 0.54
9. Were the conclusions made by the author (s) supported by the data and/or analysis reported in the overview?	76% [63.8 to 87.2]	94% [88.1–100.0]	18.9% [7.2 to 30.6] 0.01
10. How would you rate the scientific quality of this overview?	4.70 [4.47 to 4.94]	4.81 [4.59 to 5.04]	0.11 [-.28 to .70] 0.52

**Table 2:** Comparisons of percent 'yes' and percent 'differences' using the Quality of Reporting of Meta-analyses (QUOROM).

**Questions QUOROM**				**Original**	**Updated**	**Difference**
**Items**	**Heading**	*Subheading*	**Descriptor**	**Reviews yes, % reviews yes ** **[95% CI]**	**Reviews yes, % reviews yes ** **[95% CI]**	**Difference % ** **[95% CI] p-value**

**1**	**Title**		Identified the report as a meta-analysis [or systematic review] of randomized trials.	0, 0%	0, 0%	0% NS
**2**	**Abstract**		Used a structured format.	100%	100%	0% NS
**3**		*Objectives*	The clinical question explicitly.	96.2% [91.0 to 100.0]	98% [94.4 to 100.0]	1.9% [-1.8 to 7.0] 0.56
**4**		*Data sources*	The databases (e.g. list) and other information sources.	76% [63.8 to 87.2]	93% [85.3 to 99.6]	17.0% [9.8 to 28.7] 0.01
**5**		*Review methods*	The selection criteria (e.g. population, intervention, outcome, and study design; methods for validity assessment, data abstraction, and study characteristics, and quantitative data synthesis) in sufficient detail to permit replication.	40% [26.3 to 52.9]	76% [63.8 to 87.2]	35.9% [24.1 to 49.1] 0.00
**6**		*Results*	Characteristics of the randomized trials included and excluded; qualitative and quantitative findings (e.g. point estimates and confidence intervals); and subgroup analyses.	71% [59.5 to 83.9]	77% [66.0 to 88.7]	5.7% [-5.7 to 17.9] 0.50
**7**		*Conclusion*	The main results.	96% [91.4 to 100.0]	98% [94.4 to 100.0]	1.9% [-1.8 to 7.1] 0.56
**8**		*Intro*	The explicit clinical problem, biologic rationale for the intervention, and rationale for review.	98% [94.4 to 100.0]	100%	2% [1.9 to 5.6] 0.31
**9**	***Methods***	*Searching*	The information sources, in detail (e.g., databases, registers, personal files, expert informants, agencies, hand-searching], and any restrictions (e.g. years considered, publication status, language of publication).	68% [55.2 to 80.6]	87% [77.7 to 96.0]	18.9% [9.7 to 31.6] 0.02
**10**		*Selection*	The inclusion and exclusion criteria (defining population, intervention principal outcomes, and study design).	100%	96% [91.0 to 100.0]	-3.7% [-9.0 to 3.8] 0.15
**11**		*Validity assessment*	The criteria and process used [e.g., masked conditions, quality assessment and their findings.	87% [77.6 to 96.0]	96% [91.0 to 100.0]	9.4% [4.3 to 18.6] 0.08
**12**		*Data abstraction*	The process used (e.g., completed independently, in duplicate).	74% [61.6 to 85.6]	93% [85.3 to 99.6]	18.9% [11.7 to 30.9] 0.01
**13**		*Study characteristics*	The type of study design, participants' characteristics, details of intervention, outcome definitions, etc.; and how clinical heterogeneity was assessed.	89% [61.6 to 85.6]	96% [91.0 to 100.0]	7.6% [2.4 to 16.1] 0.14
**14**		*Quantitative data synthesis*	The principal measures of effect [e.g., relative risk], method of combining results (statistical testing and confidence intervals), handling of missing data, etc.; how statistical heterogeneity was assessed; a rationale for any a priori sensitivity and subgroup analyses; and any assessment of publication bias.	83% [72.8 to 93.2]	79% [68.2 to 90.2]	-3.7% [-14.8 to 6.4] 0.62
**15**	**Results**	*Trial flow*	Provide a meta-analysis profile summarizing trial flow	2% [-1.8 to 5.6]	8% [0.36 to 14.7]	5.7% [-1.5 to 9.4] 0.17
**16**		*Study characteristics*	Present descriptive data for each trial [e.g., age, sample size, intervention, dose, and duration, follow-up].	89% [80.1 to 97.3]	83% [72.9 to 93.2]	-5.7% [-15.9 to 2.9] 0.40
**17**		*Quantitative data synthesis*	Report agreement on the selection and validity assessment; present simple summary results [for each treatment group in each trial, for each primary outcome]; data needed to calculate effect sizes and confidence intervals in intention-to-treat analyses [e.g., 2 × 2 tables of counts, means and standard deviations, proportions].	81% [70.5 to 91.8]	89% [80.1 to 97.3]	7.5 [-1.1 to 18.2] 0.28
**18**	**Discussion**		Summarize the key findings; discuss clinical inferences based on internal and external validity; interpret the results in light of the totality of available evidence; describe potential biases in the review process [e.g., publication bias]; and suggest a future research agenda.	96% [91.0 to 100.0]	96% [91.0 to 100.0]	0% [-5.2 to 5.2] NS

### OQAQ

Table 1 presents the methodological quality assessments obtained using this scale. There was no significant difference in the global assessment (item 10 – How would you rate the scientific quality of the overview?) (Mean score original review 4.70, mean score updated review 4.81, difference in means 0.11 (95% CI -0.28; 0.70 p = 0.52)).

There were improvements on seven individual items, although only one item showed a significant improvement (item 9 – Were the conclusions made by the author(s) supported by the data and/or analysis reported in the overview?) (percent original reviews complying with item 76% (95% CI 4.47; 4.94) percentage updated reviews complying with item 94% (95% 88.1; 100.0) difference 18.9% (95% CI 7.2%; 30.6% p < 0.01)).

### QUOROM

Table 2 presents the assessments of reporting quality obtained using the Quality of Reviews of Meta-analyses (QUOROM) checklist. Scores (awarded for 'yes' response) on the 18 items for original Cochrane reviews ranged from 0% (item 1) to 100% (items 2 and 18). Four of the 18 items revealed improvements relating to data sources reported in the abstract (item 4) with a significant difference of 17.0% (9.8; 28.7 p = 0.01), review  methods reported in the abstract (item 5) with a difference of 35% (24.1; 49.1 p = 0.00), searching methods (item 9) with a difference of 18.9% (9.7; 31.6 p = 0.01), and data abstraction (item 12) with a difference of 18.9% (11.7; 30.9 p = 0.00).

Three questions, item (10) -3.7% (95% CI: -9.0; 3.8 p = 0.15), item (14) -3.7% (-14.8; 6.4 p = 0.62) and item (16), -5.7% (-15.8; 3.0 p = 0.40) had lower mean scores on updated reviews than on original reviews, but these differences were not statistically significant.

## Discussion

In assessing the quality of the sample of 53 systematic reviews of the Cochrane Collaboration, two assessment tools were used. The larger improvement on the QUOROM checklist than on the OQAQ suggests that although the quality of reporting has improved slightly, the quality of design and conduct has not changed. For example, the items in reporting of selection criteria and searching are improved on QUOROM, but the equivalent items on OQAQ, which relate to how well these were carried out have not changed.

The significant improvements for the OQAQ item assessing whether the conclusions drawn by the author(s) were supported by the data and/or analysis reported in the overview is also worthy of note. This could suggest that authors are paying more attention to state their conclusion in relation to the data provided. This item has been reported by other methodologists in the context of asessing quality [16,17].

Improvement in the quality of reporting of the abstract, as assessed by the QUOROM instrument, might also suggest that authors are paying more attention to the details. For example, authors might be more aware of better access to the Cochrane abstracts through electronic systems such as Medline. Also, reporting of literature searches improved. However, the literature searches themselves did not improve. This should be further explored.

Assessors expressed that they found the two instruments used in the study to have associated practical weaknesses. Their combined length (28 items) made their use somewhat cumbersome and inefficient. Another problem encountered was the lack of adequate published guidance on the application of the OQAQ question, assessing the overall methodological quality. The QUOROM checklist had the advantage of coming with fairly detailed user instructions. However, it proved very time-consuming to apply [12]. Another problem noted was that several of the questions asked by these two instruments appeared to cover the same subject matter.

The methodological and reporting quality of Cochrane reviews was fair-to-good, but further improvement is obviously needed in both areas. This was also concluded in a recent study by Moja [16]. In addition, the quality-improvement impact of updating was found to be relatively minor. On some assessed factors, particularly with respect to reporting quality, updated reviews actually scored lower than original reviews. Currently, Cochrane review updates are carried out primarily to incorporate new findings, rather than to improve quality. It would be beneficial for updates to also address reporting, such as the omission of methodological descriptions. And of course, updates should also try to improve methodological weaknesses of the reviews.

The Cochrane Collaboration endeavours to improve the quality of its systematic reviews through the application of a continuous peer review process during their development. The effectiveness of the peer-review process could probably be improved by increased attention to areas of reporting and/or methodological weaknesses. Reviewers should adhere more faithfully to the guidelines provided in the Cochrane handbook [17] in order to improve the design and conduct of reviews and to the QUOROM statement [15] to improve the quality of reporting of systematic reviews.

## Conclusion

The overall quality of Cochrane reviews is fair-to-good. Although quality of reporting improved on certain individual items there was no overall improvement seen with updating and methodological quality remained unchanged. Further improvement of quality of reporting is possible. There is room for improvement of methodological quality as well. Authors updating reviews should address identified methodological or reporting weaknesses. We recommend to give full attention to both quality domains when updating SRs.

## Competing interests

The author(s) declare that they have no competing interests.

## Authors' contributions

BS designed the protocol and the study, abstracted the data, conducted the analysis, and wrote the paper; MB designed the protocol and the study, and assisted with writing the paper; JMG provided intellectual input into the design and conduct of the study, and assisted with writing the paper; CH carried out the data abstraction, quality assessment and assisted with the analysis; LMB provided intellectual input into the design and conduct of the study, and assisted with writing the paper.

## Pre-publication history

The pre-publication history for this paper can be accessed here:



## Supplementary Material

Additional File 1 The enhanced version of the overview quality assessment questionnaire (OQAQ). Click here for file

Additional File 2 Quality of reporting of meta-analyses (QUOROM) for randomized controlled trials. Click here for file
