# Supplementary material for: Does updating improve the methodological and reporting quality of systematic reviews?
Source: BMC Med Res Methodol. 2006 Jun 13;6:27. doi: 10.1186/1471-2288-6-27 (PMC1569863; doi:10.1186/1471-2288-6-27)
Supplement: Additional File 1 — The enhanced version of the overview quality assessment questionnaire (OQAQ). [file 1471-2288-6-27-S1.doc]

The purpose of this index is to evaluate the scientific quality (i.e. adherence to scientific principles) of research overviews (review articles) published in the medical literature. It is not intended to measure literary quality, importance, relevance, originality, or other attributes of overviews.

The index is for assessing overviews of primary (“original”) research on pragmatic questions regarding causation, diagnosis, prognosis, therapy or prevention. A research overview is a survey of research. The same principles that apply to epidemiologic surveys apply to overviews: a question must be clearly specified, a target population identified and accessed, appropriate information obtained from that population in an unbiased fashion, and conclusions derived, sometimes with the help of difference between overviews and epidemiologic surveys the unit of analysis, not the scientific issues that the questions in this index address.

Since most published overviews do not include a methods section it is difficulty to answer some of the questions in the index. Base you answer, as much as possible, on the information provided in the overview. If the methods that were used are reported incompletely relative to a specific item, score that item as “partially”. Similarly, if there is no information provided regarding what was done relative to a particular question, score it as “can’t tell”, unless there is information in the overview to suggest either that the criterion was or was not met.

1. Were the search methods used to find evidence (original research) on the primary question (s) stated?

 yes  partially  no

Yes is given to meta-analysis reporting categories of sources, including years (e.g., databases-Medline) used, and whether these categories were named (e.g. Medline). Partial points are given for the category of sources (e.g., electronic, hand, register) are named.

1. Was the search for evidence reasonably comprehensive?

 yes  can’t tell  no

Yes is given if at least three categories, one of which must be electronic with key words stated, and any two others (e.g., hand, register) are reported. Key words and/or MESH terms must be stated.

1. Were the criteria used for deciding which studies to include in the overview reported?

 yes  partially  no

This item was thought to be reasonably explicit. If 2 or more items mentioned, yes, if <2 mentioned, partially, if none mentioned, no.

1. Was bias in the selection of studies avoided?

 yes  can’t tell  no

Yes is given if at least two reviewers independently assess for inclusion. A consensus must be reached.

1. Were the criteria used for assessing the validity of the included studies reported?

 yes  partially  no

It was felt that the issues relating to publication bias should not be included in the assessment of this. Yes is given to those meta-analysis reporting ‘a priori’ methods of validity assessment (e.g., if the author(s) chose to include only randomized, double-blind, placebo controlled trials, or allocation concealment as inclusion criteria).

1. Was the validity of all studies referred to in the text assessed using appropriate criteria (either in selecting studies for inclusion or in analysing the studies that are cited)?

 yes  can’t tell  no

This item relates to validity assessment. Yes is given if there is a description of any criteria (either internal or external) used either for inclusion, or for analysis (e.g., sensitivity analysis).

1. Were the methods used to combine the findings of the relevant studies (to reach a conclusion) reported?

 yes  partially  no

This item was thought to be reasonably explicit.

1. Were the findings of the relevant studies combined appropriately relative to the primary question the overview addresses?

 yes  can’t tell  no

For question 8, if no attempt was made to combine findings, and no statement is made regarding the inappropriateness of combining findings, check “no”. If a summary (general) estimate is given anywhere in the abstract, the discussion or the summary section of the paper, and it is not reported how the estimate was derived, mark “no” even if there is a statement regarding the limitations of combing the findings of the studies reviewed. If in doubt mark “can’t tell”.

1. Were the conclusions made by the author(s) supported by the data and/or analysis reported in the overview?

 yes  partially  no

For an overview to be scored as “yes” on question 9, data (not just citations) must be reported that supports the main conclusions regarding the primary questions (s) that the overview addresses. If the overview concerns diagnostic/prognostic tests, ‘retest is not required’ (this ensures that diagnostic/prognostic papers are not scored more rigorously than clinical papers).

1. How would you rate the scientific quality of the overview?

Extensive Major Minor Minimal
Flaws Flaws Flaws Flaws

      

1 2 3 4 5 6 7

The score for question 10, the overall scientific quality, should be based on your answers to the first nine questions. The following guidelines can be used to assist with deriving a summary score. If the “can’t tell” option is used one or more times on the preceding questions, a review is likely to have minor flaws at best and it is difficult to rule out major flaws (i.e. a score of 4 or lower). If the “no” option is used on question 2, 4, 6 or 8, the review is likely to have major flaws (i.e. a score of 3 or less, depending on the number and degree of the flaws).
