# Supplementary material for: Does updating improve the methodological and reporting quality of systematic reviews?
Source: BMC Med Res Methodol. 2006 Jun 13;6:27. doi: 10.1186/1471-2288-6-27 (PMC1569863; doi:10.1186/1471-2288-6-27)
Supplement: Additional File 2 — Quality of reporting of meta-analyses (QUOROM) for randomized controlled trials. [file 1471-2288-6-27-S2.doc]

| **Heading** | **Descriptor** | **Reported**  [Y/N] | **Page #** |
| --- | --- | --- | --- |
| **Title** | Identify the report as a meta-analysis [or systematic review] of randomized trials. |  |  |
| **Abstract** | Use a structured format. |  |  |
| *Objectives* | The clinical question explicitly. |  |  |
| *Data sources* | The databases [i.e., list] and other information sources. |  |  |
| *Review methods* | The selection criteria [i.e., population, intervention, outcome, and study design]; methods for validity assessment, data abstraction, and study characteristics, and quantitative data synthesis] in sufficient detail to permit replication. |  |  |
| *Results* | Characteristics of the randomized trials included and excluded; qualitative and quantitative findings [i.e., point estimates and confidence intervals]; and subgroup analyses. |  |  |
| *Conclusion* | The main results. |  |  |
| **Introduction** | The explicit clinical problem, biologic rationale for the intervention, and rationale for review. |  |  |
| **Methods** |  |  |  |
| *Searching* | The information sources, in detail [e.g., databases, registers, personal files, expert informants, agencies, hand-searching], and any restrictions [years considered, publication status , language of publication. |  |  |
| *Selection* | The inclusion and exclusion criteria [defining population, intervention principal outcomes, and study design]. |  |  |
| *Validity assessment* | The criteria and process used [e.g., masked conditions, quality assessment and their findings. |  |  |
| *Data abstraction* | The process used [e.g., completed independently, in duplicate]. |  |  |
| *Study characteristics* | The type of study design, participants' characteristics, details of intervention, outcome definitions, etc.; and how clinical heterogeneity was assessed. |  |  |
| *Quantitative data synthesis* | The principal measures of effect [e.g., relative risk], method of combining results [statistical testing and confidence intervals], handling of missing data, etc.; how statistical heterogeneity was assessed; a rationale for any a priori sensitivity and subgroup analyses; and any assessment of publication bias. |  |  |
| **Results** |  |  |  |
| *Trial flow** | Provide a meta-analysis profile summarizing trial flow [figure]. |  |  |
| *Study characteristics* | Present descriptive data for each trial [e.g., age, sample size, intervention, dose, duration, follow-up]. |  |  |
| *Quantitative data synthesis* | Report agreement on the selection and validity assessment; present simple summary results [for each treatment group in each trial, for each primary outcome]; data needed to calculate effect sizes and confidence intervals in intention-to-treat analyses [e.g., 2 x 2 tables of counts, means and standard deviations, proportions]. |  |  |
| **Discussion** | Summarize the key findings; discuss clinical inferences based on internal and external validity; interpret the results in light of the totality of available evidence; describe potential biases in the review process [e.g., publication bias]; and suggest a future research agenda |  |  |

Potentially relevant RCTs identified and screened for retrieval [n = ...]

RCTs excluded, with reasons [n = ...]

RCTs retrieved for more detailed evaluation [n = ...]

RCTs excluded, with reasons [n = ...]

Potentially appropriate RCTs to be included in the meta-analysis [n = ...]

RCTs included in meta-analysis [n = ...]

RCTs excluded from the meta-analysis, with reasons [n = ...]

RCTs with usable information, by outcome [n = ...]

RCTs withdrawn, by outcome, with reasons [n = ...]

#### *Progress through the stages of a meta-analysis, including selection of potentially relevant randomized controlled trials [RCTs], included and excluded RCTs with a statement of the reasons, RCTs with usable information, and RCTs withdrawn by outcome with a statement of the reasons for the withdrawal.

Moher D, Cook DJ, Eastwood S, Olkin I, Rennie D, Stroup DF: **Improving the quality of reports of meta-analyses of randomized controlled trials: the QUOROM statement. Quality of reporting of meta-analyses.** Lancet 1999, **354**: 1896-900.
